# Evidence of mesenchymal stromal cell adaptation to local microenvironment following subcutaneous transplantation

**DOI:** 10.1111/jcmm.15717

**Published:** 2020-08-12

**Authors:** Mihai Bogdan Preda, Ana‐Mihaela Lupan, Carmen Alexandra Neculachi, Livia Ioana Leti, Ioana Madalina Fenyo, Sinziana Popescu, Evelyn Gabriela Rusu, Catalina Iolanda Marinescu, Maya Simionescu, Alexandrina Burlacu

**Affiliations:** ^1^ Institute of Cellular Biology and Pathology 'Nicolae Simionescu' Bucharest Romania

**Keywords:** angiogenesis, hypoxia, mesenchymal stromal cells, remote activity, subcutaneous transplantation

## Abstract

Subcutaneous transplantation of mesenchymal stromal cells (MSC) emerged as an alternative to intravenous administration because it avoids the pulmonary embolism and prolongs post‐transplantation lifetime. The goal of this study was to investigate the mechanisms by which these cells could affect remote organs. To this aim, murine bone marrow–derived MSC were subcutaneously transplanted in different anatomical regions and the survival and behaviour have been followed. The results showed that upon subcutaneous transplantation in mice, MSC formed multicellular aggregates and did not migrate significantly from the site of injection. Our data suggest an important role of hypoxia‐inducible signalling pathways in stimulating local angiogenesis and the ensuing modulation of the kinetics of circulating cytokines with putative protective effects at distant sites. These data expand the current understanding of cell behaviour after subcutaneous transplantation and contribute to the development of a non‐invasive cell‐based therapy for distant organ protection.

## 1 INTRODUCTION

Although clear progress has been achieved in the regenerative medicine field during the past 20 years, effective regeneration of diseased organs is still a challenge and demands new therapeutic strategies.^1^ Mesenchymal stromal/stem cells (MSC) residing in almost all postnatal organs and tissues^2^, ^3^ are an attractive option for tissue repair, owing to their ability to protect from apoptosis, stimulate the angiogenesis,^4^ support the stromal remodelling^5^ and modulate the immune responses.^6^, ^7^

Experimental studies have shown that intravenous (*iv*) transplantation of MSC prevented the progress of a wide array of diseases, including cardiovascular or autoimmune diseases^8^, ^9^, ^10^; however, the mechanism involved has not been completely deciphered. Moreover, it should be noted that the intravascular infusion, which is the most popular route for MSC delivery in both pre‐clinical studies and clinical trials, comes with the risk of adverse events, for example pulmonary embolism.^11^, ^12^ The general understanding is that MSC have a short lifespan after *iv* administration, and experimental evidence showed that, soon after transplantation, the majority of administered cells are trapped in the lung capillaries.^13^ Nonetheless, *iv* infusion of MSC was reported to reduce the inflammatory response and promote tissue repair^14^, ^15^ in many experimental settings, which indicated an important role of the secretome (MSC‐secreted molecules) in modulating the innate and adaptive immune responses.^6^, ^14^, ^16^

Based on the reported therapeutic effects of the MSC secretome, we and others have proposed the subcutaneous transplantation procedure as an alternative to *iv* administration of MSC, the benefit of which is to overcome the risk of pulmonary embolism and prolong the lifetime of cells post‐transplantation.^17^, ^18^, ^19^, ^20^

Here, we provide evidence that after subcutaneous transplantation, MSC shape into multicellular aggregates that activate hypoxia signalling pathways and the ensuing local angiogenesis. This is followed by the transient modulation of a large panel of circulating cytokines with putative protective effects at distant sites. These data sustain the existence of a blood‐borne–mediated pathway activated by MSC after subcutaneous transplantation, with no need of homing to the site of injury.

## 2 MATERIALS AND METHODS

### 2.1 Animals

All animal experiments were conducted in accordance with the European Guidelines for Animal Welfare (Directive 2010/63/EU) and approved by the National Sanitary Veterinary and Food Safety Authority (nr 390/10/07/2018). C57BL/6J mice were purchased from the Jackson Laboratory and bred in the animal facility of the Institute of Cellular Biology and Pathology under specific pathogen‐free conditions in a controlled environment of 12/12‐hour light/dark cycle, 21°C and 55%‐60% humidity, with chow and water ad libitum.

### 2.2 Isolation and characterization of MSC

The cells were isolated from mouse bone marrow as previously described.^4^ Briefly, bone marrow was obtained from male C57BL/6 mice of 6‐8 weeks of age by flushing the medullary cavity of femurs and tibias with complete medium, consisting in low‐glucose DMEM, supplemented with 10% MSC‐qualified FBS and 1% antibiotic‐antimycotic (all reagents were purchased from Thermo Fisher Scientific). Then, the cell suspension was passed through needles of decreasing size from 18 to 25 gauge to obtain a single cell suspension. Collected cells were centrifuged at 400 *g* for 5 minutes, resuspended in complete medium and seeded at 10^6^ cells/cm^2^. At 24 hours, the non‐adherent cells were removed by changing the medium. After 1 week, the cells were detached with 0.25% trypsin and gently scraped with a rubber policeman, followed by seeding at a density of 5000 cells/cm^2^ in complete medium. The next 5‐6 passages were done at 90% confluency, until the culture was totally free of CD45^+^ cells (starting at passage no 7). The presence of MSC characteristic markers (Sca‐1, CD105, CD44), the absence of haematopoietic markers CD45 and CD11b, and the in vitro differentiation potential of cells into osteogenic, adipogenic and chondrogenic lineages were evaluated to confirm the MSC attributes.^4^ These attributes were retained for at least 10 passages after completing the selection process.^21^ Cells were used between the 8th and 13th passages. The 3D aggregates were obtained by assembling various number of cells (from 10^4^ to 3 × 10^5^) for 3 days using the hanging‐drop method as previously described.^22^ The aggregate diameter was determined under a Nikon Eclipse Ti‐E inverted microscope using a Ds‐Fi1 camera (Nikon) and NIS‐Elements AR 3.0 software.

Cell survival and proliferation was monitored in vivo, after transfection with pLNC‐Luc plasmid, and 3‐week selection with Geneticin (500 µg/mL). To obtain pLNC‐Luc plasmid, luciferase gene was cloned from pGL3‐Basic plasmid (Promega) into pLNCX2 plasmid (Clontech) using ClaI and HindIII restriction enzymes.

For in vivo imaging of hypoxia, the cells were transfected with HRE‐luciferase plasmid (Addgene # 26731, a gift from Navdeep Chandel)^23^ or miR‐210 promoter‐Luc construct (a kind gift from Dr Fabio Martelli),^24^ by electroporation (NEPA21; Nepagene), 24 hours prior to injection. In addition, for in vivo tracking, in some experiments MSC were fluorescently stained with Red CMTPX Dye (Thermo Fisher Scientific), according to the manufacturer's recommendations.

### 2.3 MSC transplantation procedure

Mice were anaesthetized with a mixture of ketamine‐xylazine (100‐5 mg/kg bodyweight), and the hair around the site of injection was removed with an electrical clipper. To elect the most favourable place for subcutaneous MSC injection, 50 µL of cell suspension (containing 10^6^ or 3.5 × 10^6^ cells) was slowly injected subcutaneously in different anatomical regions (interscapular, inguinal and abdominal). This way, the cell spreading was avoided and the formation of 3D aggregate was certified by the presence of the subcutaneous swelling after transplantation.

### 2.4 Assessment of serum cytokines after MSC subcutaneous transplantation

The serum profile of sham‐ and MSC‐treated mice was analysed using a Proteome Profiler Mouse XL Cytokine Array Kit (ARY028; R&D Systems). Thus, three doses of 10^6^ MSC/50 µL PBS were injected subcutaneously in each animal (n = 3) in the three anatomical regions mentioned above. Blood samples collected before and at 1, 3 and 5 days after cell transplant were pooled from three animals in each group for each time‐point. The chemiluminescence produced by each spot was measured with a GE Healthcare ImageQuant LAS‐4000 Analyser, and the pixel density was quantified by TotalLab CLIQS software. Results were analysed using the online software Morpheus (https://software.broadinstitute.org/morpheus), in order to compact bulky information into clusters and find patterns in the data.

### 2.5 In vitro assays for visualization of hypoxia

Cells were cultured in hanging drops in culture medium supplemented with 100 nmol/L HypoxiSense 680 (HS680) from PerkinElmer. Various sizes of cellular aggregates were obtained by seeding different cell numbers (10^3^, 5 × 10^3^, 10^4^, 2 × 10^4^ and 5 × 10^4^) per hanging drop. After 3 days, cellular aggregates were briefly washed three times with PBS and imaged with IVIS Spectrum system and Living Image 4.5 software (PerkinElmer) to measure the fluorescent signal. Spectral unmixing analysis was performed to extract the autofluorescence and HS680 fluorescence signal was calculated as total radiant efficiency.

Alternatively, MSC seeded as monolayers in Nunc™ Lab‐Tek™ II Chambered Coverglass (Thermo Fisher Scientific) were incubated with 100 nmol/L HS680 in complete medium for 24 hours. Subsequently, the cells were washed three times with PBS and live‐imaged with a confocal microscope (Leica TCS‐SP5) using a HeNe 633 laser (PMT emission bandwidth of 40 nm: 670‐710 nm) and a PL APO 10×/0.4 NA Dry CS objective. Images were acquired using identical acquisition settings and pinholes of 1 Airy Unit.

### 2.6 In vivo assays for cell migration, hypoxia visualization and determination of the inflammatory response

Cell migration was evaluated using CMTPX‐labelled cells grafted subcutaneously in different anatomical regions (described above), and the animals were imaged at 30 minutes and at 7 days after cell injection using the IVIS Spectrum. At the time of harvest, the skin around transplanted cells and different organs were analysed ex vivo, in order to more precisely evaluate cell survival and identify putative sites of cell homing.

Hypoxia visualization in vivo was performed by two methods: (a) transplantation of unlabelled cells followed by *iv* injection of HS680 (1.4 nmol in 200 µL PBS) 24 hours before IVIS analysis, or (b) transplantation of MSC labelled with HRE‐Luc or miR‐210 promoter‐Luc construct and injecting intraperitoneally D‐luciferin (130 mg/kg b.wt.) 10 minutes before imaging.

To determine the local inflammatory response in vivo after MSC transplantation, animals were injected *i.p*. with lucigenin (12.5 mg/kg body weight) 10 minutes before imaging, as previously described.^25^ Surface images were analysed using Living Image 4.5 software (PerkinElmer) and quantification of bioluminescence was done by manually defining the regions of interest. The bioluminescent signal was calculated as average radiance.

### 2.7 Statistics

The results were expressed as the mean ± SEM (standard error of the mean). Statistical analyses were performed using GraphPad Prism 7.0 (GraphPad Software, Inc). Comparisons between groups were done using unpaired *t* tests with two‐tailed distribution or two‐way ANOVA using a Bonferroni post hoc test where appropriate. Difference was considered statistically significant when *P*‐value was <.05.

## 3 RESULTS

### 3.1 Establishing the most favourable transplantation site and dose for the MSC graft stability

The first issue addressed was to select the site of MSC subcutaneous transplantation that offers the maximum benefits, in terms of local cell survival and engraftment. To this purpose, CMTPX‐labelled MSC were subcutaneously transplanted in three different anatomic regions, each of them receiving different inputs from the nearby adipose tissue: interscapular (in the proximity of brown adipose tissue), inguinal (in the proximity of white adipose tissue) and abdominal (with no adipose tissue nearby) (Figure S1a). The animals were imaged at 30 minutes and at 7 days after cell transplantation. The fluorescent signal indicated no significant differences between the three groups (Figure S1b); moreover, the intensity determined at 7 days after transplantation was with one order of magnitude lower than that of the signal quantified 30 minutes after the procedure. The data indicated that similar number of cells was engrafted and survived locally when transplanted in either region, and there was no influence of the nearby adipose tissue.

**FIGURE 1:**
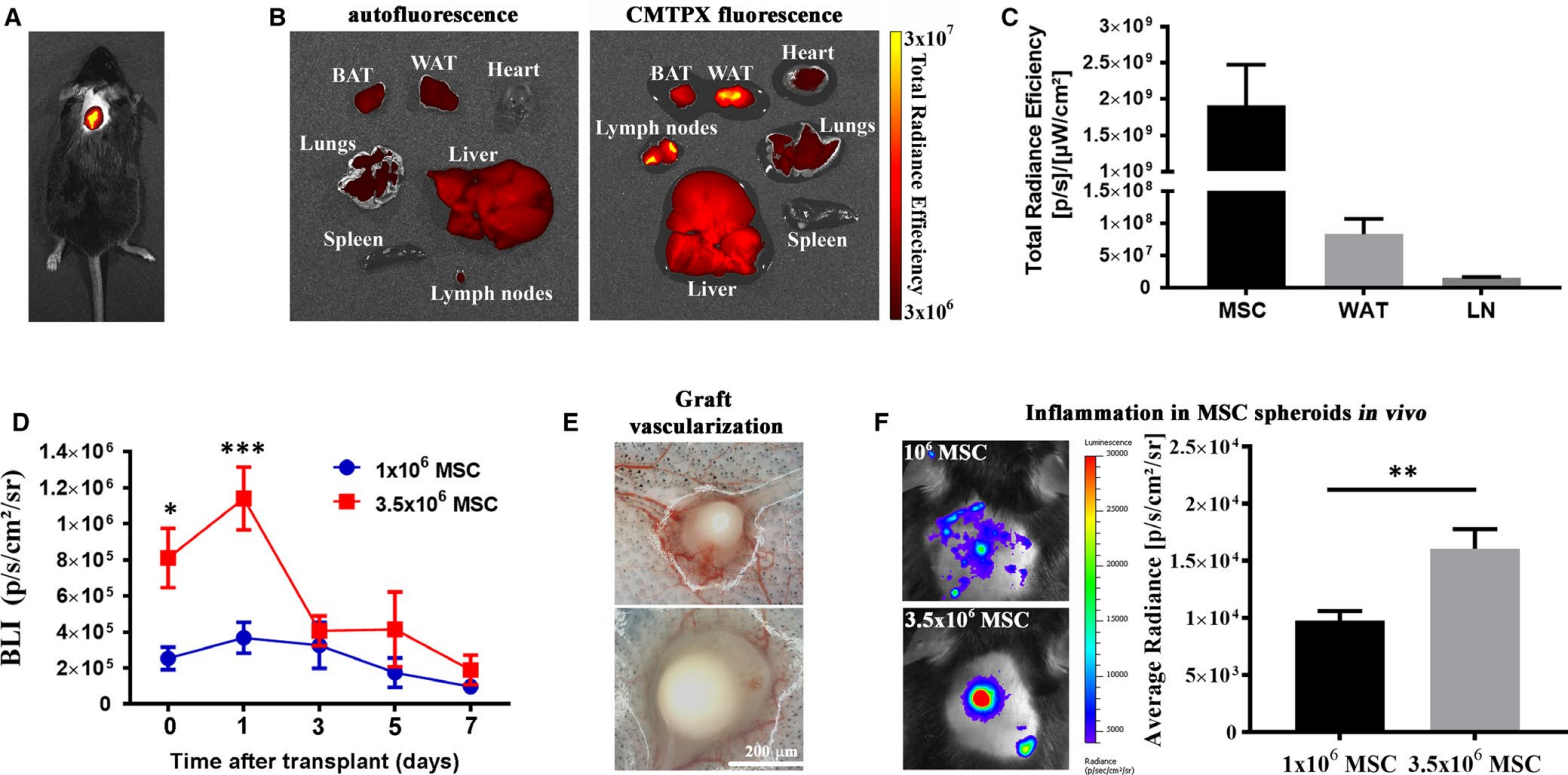
Behaviour of MSC after subcutaneous transplantation in the interscapular region. A, Fluorescent signal of the labelled MSC graft 30 min after the transplantation, as assessed by in vivo imaging. B, representative ex vivo fluorescence imaging of mouse organs at 7d post‐transplantation of CMTPX‐labelled MSC (right) or with vehicle, as control (left); BAT, brown adipose tissue; WAT, white adipose tissue. C, total radiant efficiency values of spectral‐unmixed signal in the MSC aggregate, WAT and lymph node (LN) at 7d after transplantation. D, survival curve for two cell doses (low and high) of grafted cells determined by the in vivo bioluminescent (BLI) signal of Luc‐expressing MSC measured as average radiance. E, representative images of in situ vascularization of MSC aggregates at 7d post‐transplant (after skin excision and graft exposure). F, in vivo assessment of graft‐induced inflammation by quantification of myeloperoxidase‐based bioluminescent signal after i.p. injection of lucigenin in mice receiving low and high MSC doses (**P < .01, Student's *t* test)

To evaluate whether and to which extent MSC migrated from the site of transplantation, analysis of the major organs involved in cell migration was performed at the time of harvest by ex vivo fluorescence imaging. Of the analysed organs (spleen, lymph nodes, adipose tissue, lung, liver and heart), only local lymph nodes and adjacent adipose tissue showed detectable, although very low, fluorescent signals in all groups, which were around two orders of magnitude lower than that of the locally transplanted cells (Figure S1c). For instance, at 7 days after subcutaneous transplantation of MSC in the interscapular region, 4.4 ± 2.4% and 0.8 ± 0.4% of the graft signal were detected in the interscapular adipose tissue and lymph nodes, respectively, suggesting that MSC did not significantly migrate from the injection site (Figure 1A‐C). Based on these data, the interscapular site was selected for the subsequent experiments of MSC transplantation, a route that was also used in our previous work.^17^

The next issue we addressed was the optimal cell number to be transplanted. To assess whether cell number has an impact on the survival of the cells in vivo, a low dose (1 × 10^6^ cells) and a high dose (3.5 × 10^6^ cells) of Luc‐expressing MSC were subcutaneously transplanted into the interscapular region and the bioluminescent signal was measured at different times after transplantation, as a direct method to comparatively estimate the cell survival in the assembled aggregates. One day after transplantation, the results showed a transient increase in bioluminescent signal, for both cell doses, possibly an effect of cell recovery after in vitro manipulations; this was followed by a gradual reduction of the signal (Figure S2). It is worth mentioning that the total BLI signal is influenced by multiple factors, such as luciferin penetrance into the aggregate, cellular status, oxygen availability, cell proliferation and cell death. However, the decrease in survival sloped faster in high‐dose cell aggregate than in low‐dose cell aggregate (Figure 1D and S2); at 7 days after transplantation, the cell survival rates were 57% ± 12% in low dose and 17% ± 6% in high dose. In terms of estimated cell number, the two aggregates contained the same number of viable cells at 7 days after transplantation, as the bioluminescent signals from the two groups were similar, irrespective of the initial cell dose (Figure 1D). These results demonstrated that the cell viability is a constraining factor in establishing the transplantation dose, being likely governed by the competition for microenvironmental nutrients. Moreover, low‐dose cell aggregates showed a greater vessel ingrowth at day 7 (Figure 1E), indicating that cell survival depends on the presence of a stable host‐derived vascular network to support the biological functions of the grafted cells.

To further assess whether the grafted cells elicit local inflammation, animals were injected with lucigenin (12.5 mg/kg body weight, *i.p*.) at 7 days after transplantation and the presence of macrophages within the grafts was assessed based on the bioluminescence signal produced by the direct interaction between phagocyte NADPH oxidase and lucigenin. The results revealed that the bioluminescent signal produced in low‐dose aggregates was close to background level, which indicated low inflammation associated with the MSC grafts; however, an increased local inflammatory bioluminescent signal was noted in high‐dose cell aggregates (Figure 1F). Together, the above data revealed that the transplantation dose, but not the transplantation site, is a limiting factor of graft stability. The subcutaneously transplanted MSC are not affected by the local adipose tissue and do not considerably migrate from the injection site, and the graft survival is enhanced when using low number of cells, which facilitates the growth of vascular network and the ensuing access of nutrients, while limiting local inflammation.

### 3.2 Hypoxia is activated in MSC aggregates both in vitro and in vivo

**FIGURE 2:**
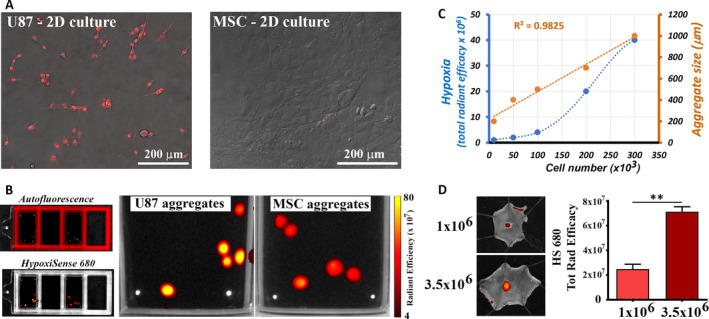
HS680 fluorescent imaging agent labels hypoxic MSC in large aggregates. A, Merged confocal images of DIC and fluorescence of U87‐MG cells and MSC incubated with HS680 for 24 h in 2D culture. B, spectral unmixing analysis of 3D multicellular aggregates of U87‐MG cells and MSC cultivated for 3 d in hanging drops in the presence of HS680 dye. This analysis was performed using the IVIS Spectrum system and Living Image 4.5 software, and allowed to separate the autofluorescence signal from the specific signal of HS680 dye. C, The correlation between aggregate diameter and hypoxia level in MSC spheroids formed by hanging‐drop culture of increasing numbers of MSC. The values are mean ± SEM of at least 10 spheroids per experimental condition. D, quantification of hypoxia of in vivo MSC aggregates at 7 d after subcutaneous injection of 1 × 10^6^ or 3.5 × 10^6^ cells (measured as HS680‐specific signal). Data are shown as mean ± SEM of n = 5 mice. (**P < .01, Student's t test)

Subcutaneously transplanted MSC engrafted and survived only if vascularized, and this process was particularly evident when low numbers of cells were employed. In an attempt to identify the mechanisms associated with graft vascularization in low‐dose MSC aggregates formed after subcutaneous transplantation, we questioned whether activation of hypoxia could play a role in this process. To this aim, the binding of HS680, a fluorescent imaging agent that detects the cell surface expression of carbonic anhydrase 9 (CA9),^26^ to the surface of hypoxic cells was first checked on MSC, in vitro. Our data showed that, in contrast to U87‐MG tumour cell line, which reportedly exhibit a strong constitutive expression of CA9 protein,^27^ MSC did not bind HS680 in normoxic 2D culture conditions (Figure 2A). In contrast, HS680 specifically and strongly bound to MSC aggregates (formed by hanging‐drop assay) at a comparable level to U87‐MG cells (Figure 2B). Besides, CA9 protein level was significantly increased in 2D‐cultured MSC exposed to hypoxic conditions (Figure S3), thus confirming the capacity of HS680 to label hypoxic MSC.

Assembling of different numbers of MSC in the hanging‐drop assay in the presence of HS680 revealed a linear correlation between the cell number and the aggregate diameter size (*R* ^2^ = 0.9825). Yet, a monotonic correlation was detected between the cell number and hypoxia level, namely higher hypoxia signals in larger aggregates (Figure 2C). These data sustain the existence of a threshold value for the aggregate size above which the hypoxia signalling is activated within the aggregate, an observation that correlates well with other reports.^28^, ^29^ Thus, aggregates formed by less than 10^5^ cells exhibit only mild levels of hypoxia, while in aggregates formed by more than 2 × 10^5^ cells, the increase in hypoxia signal rate was significantly higher.

These results were further investigated by in vivo experiments, in which mice were transplanted with either a high dose (3.5 × 10^6^) or a low dose (1 × 10^6^) of MSC (Figure 2D). After 2 and 7 days, the animals were *iv* injected with HS680 and the fluorescent signal of the aggregates was assessed, first in vivo, and then ex vivo, by imaging the inner face of the skin containing the aggregate. While the signal could not be detected in living animals (data not shown), specific HS680 signal was detected in cell aggregates ex vivo, at 7 days after transplantation (Figure 2D). Although both doses resulted in hypoxia activation, higher signals were measured in high‐dose cell aggregates as compared to low‐dose cell aggregates. Importantly, no specific HS680 signal was detected at 2 days after transplantation, either in vivo or ex vivo (data not shown), suggesting that HS680 could only detect prolonged and/or severe hypoxia in MSC aggregates.

### 3.3 Time‐course activation of hypoxia signalling pathways in vivo

**FIGURE 3:**
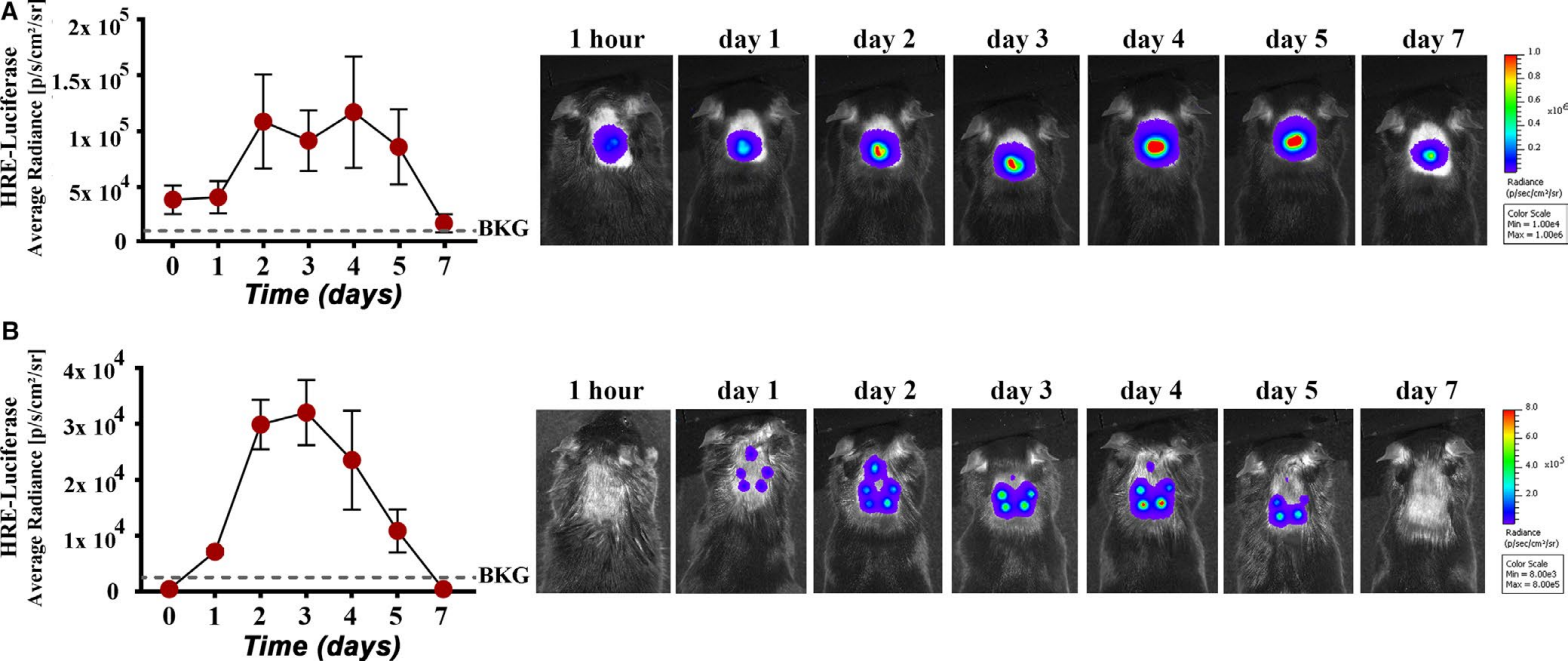
Subcutaneously transplanted MSC activate hypoxia signalling pathways. A, Time‐course evaluation of hypoxia activation in HRE‐Luc‐expressing MSC when 10^6^ cells were transplanted subcutaneously in mice (n = 6). Representative images of one mouse at different time‐points after transplant are illustrated on the right. B, time‐course evaluation of hypoxia activation when five doses of 2 × 10^5^ HRE‐luciferase‐expressing MSC were injected subcutaneously in different adjacent sites in a mouse (n = 4). The representative images of one mouse at different time‐points after transplant are illustrated on the right

Hypoxia was assessed in MSC grafts by following the time‐course activation of HIF‐1α signalling pathway in transplanted cells. To this aim, MSC were transiently transfected with a HRE‐luciferase construct before being subcutaneously transplanted. Transfection efficiency, evaluated using pEGFP‐N1 vector (Clontech) and flow cytometry analysis, was more than 60% at 24 hours post‐transfection (Figure S4). In vivo bioluminescence analysis of transplanted mice showed that hypoxia signalling was activated 1 day after cell transplantation and persisted for at least four days (Figure 3A), thus suggesting a time window for hypoxia signalling in MSC of several days following subcutaneous transplantation.

To assess whether hypoxia activation is an intrinsic mechanism and not a high‐dose–induced effect, the same cell number (10^6^ cells) was administered as five distinct injections forming rosette‐shaped aggregates, so that each of the five cell aggregates consisted of one fifth of the total cell number. The results were very much the same as those with single injections, with activation of HIF‐1α signalling pathway starting on day 1 post‐transplant and maintained for 4 days (Figure 3B). As hypoxia‐induced HIF activation is reportedly followed by miR‐210 induction, as a common feature of the hypoxic response in cancer and normal cells,^24^, ^30^ the promoter activation of miR‐210 was also evaluated in MSC aggregates in vivo. By using a similar luciferase assay, the results showed the activation of miR‐210 between days 1 and 4 after subcutaneous transplantation of MSC (Figure S5). Together, these data suggested that hypoxia signalling is important in grafted cell aggregates for vascular network infiltration and graft stabilization. Our data are in agreement with the results showing that HIF‐1a is a master regulator of angiogenesis, participating in vascular formation by synergistic correlations with other pro‐angiogenic factors such as VEGF, PDGF, FGF and angiopoietins.^30^

### 3.4 Candidate biomolecules involved in the remote protective effects of MSC

**FIGURE 4:**
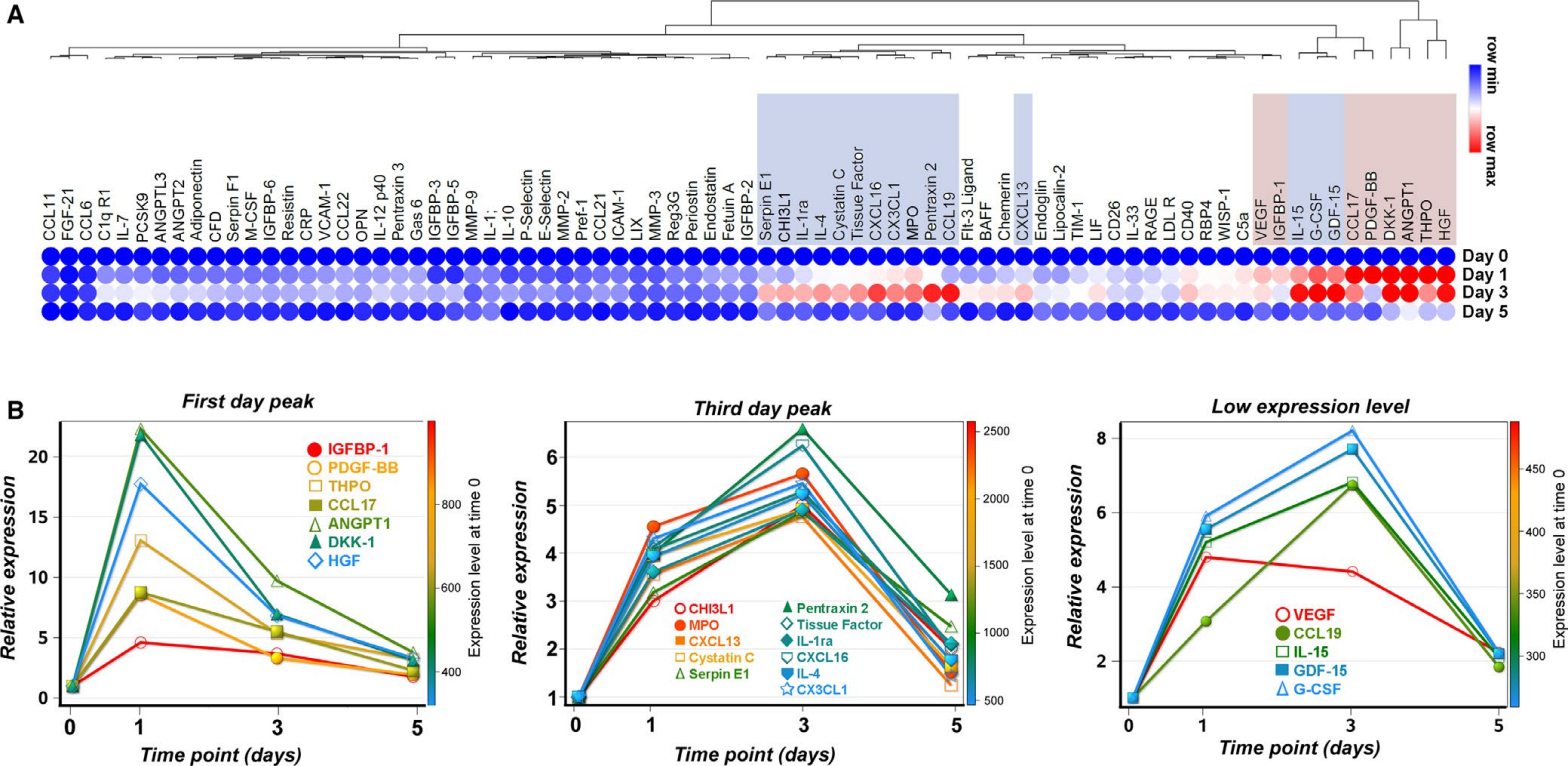
Dynamic changes in the cytokine abundance in the mouse serum after subcutaneous transplantation of MSC. A, Hierarchically clustered heat map showing the Euclidian distance of serum cytokines differentially expressed at various time‐points after subcutaneous transplantation of MSC in mice. Values represent fold change relative to day 0 and are adjusted to a per row colour scale. The cytokines with first‐day peak level are highlighted in magenta, while cytokines with third‐day peak level are highlighted in violet. B, Graphs illustrating the highly expressed cytokines clustered according to their max peak expression in the serum. At right, the “low expression level” denotes the cytokines whose maximum level of expression at any time‐point was higher than the half but below two thirds of the median value of all spots (highest pixel density between 2000 and 2500). The *y*‐axis on the left represents the relative expression level as fold change as compared to day 0. The colour‐coded right *y*‐axis shows the abundance of each cytokine in the serum of mice before transplant (day 0)

As MSC do not considerably migrate from the site of subcutaneous transplantation, the locally assembled aggregates could have remote protective activities in injured organs via the release of circulating mediators. To address this issue, proteome profiler cytokine array was performed from mouse serum collected before and at 1, 3 and 5 days after subcutaneous transplantation of MSC. Surprisingly, transplantation of MSC resulted in a broad, yet transient, effect on the cytokine profile in the circulation of transplanted animals (Figure 4A). Matrix analysis after hierarchical clustering revealed a group of molecules that were highly increased in the serum of transplanted animals (Figure 4A). Part of these proteins, namely angiopoietin‐1, DKK‐1, HGF, thrombopoietin, CCL17, PDGF‐BB and VEGF, had peak levels (between 4.5‐ and 22‐fold change) 1 day after transplant, while others, that is MPO, fractalkine, CXCL16, tissue factor, pentraxin‐2, cystatin, IL‐4, IL‐1ra, CXCL13, serpin E1, chitinase 3‐like 1, CCL19, IL‐15, GDF‐15 and G‐CSF, had peak levels (higher than 4.5‐fold change) at 3 days after transplant (Figure 4B). Among these cytokines, IL‐1ra and IL‐4 have also been previously reported as being highly secreted by MSC in culture, in both normoxic and hypoxic conditions.^4^ Moreover, IL‐1ra was also identified in the secretome of MSC, but not of dermal fibroblasts, aggregated in hanging‐drop culture, which thus qualified it as a putative MSC‐derived cytokine responsible for the effector properties in vivo (Figure S6). It is also worth mentioning that pentraxin‐3, previously identified as increased in MSC aggregates in vivo and in the serum of transplanted mice,^17^ was identified as having a threefold increase in serum collected at 1 and 3 days after transplantation (Figure 4A). These results strengthen the hypothesis of MSC‐released circulating mediators to induce tissue regeneration in remote diseased organs. More experiments are needed to find the most important biomolecules involved in this process.

## 4 DISCUSSION

The results of our experiments revealed that upon subcutaneous transplantation, MSC do not significantly migrate from the graft and organize into multicellular aggregates. These aggregates activate hypoxia‐inducible signalling pathways, which in turn stimulate local angiogenesis and a broad release of therapeutic biomolecules into the circulation, a mechanism that can contribute to their therapeutic effects on remote organs.

Although intravascular administration is the most popular delivery route in MSC‐based therapy, it offers only minimal engraftment of the cells into recipient organs, a feature that can explain the mixed clinical outcomes reported before.^31^, ^32^ At the same time, the concept of remote therapy is being increasingly recognized in the research field. We have previously showed that subcutaneous transplantation of MSC protected the heart against ischaemia‐reperfusion injury in mice,^17^ whereas Shabbir et al^19^ reported that delivery of MSC into the skeletal muscle bed improved ventricular function in a hamster heart failure model. In good agreement with our data, Mao et al^33^ have recently reported that intramuscular injection of human MSC improved cardiac function in dilated cardiomyopathy in rats, possibly by regulation of relevant cytokines in the serum and in the myocardium. Other data supported the idea that remote transplantation of MSC represented an alternative to *iv* infusion, ensuring an extended cell survival.^11^ All these results are in favour of remote MSC therapy as a minimally invasive strategy that can provide sustained delocalized benefits for diverse applications.

Taking advantage of the ability of MSC to secrete molecules that operate in an endocrine‐like manner, we put forward the subcutaneous (remote) transplantation as a therapeutic approach that offers several advantages as compared to other routes of cell delivery. First, the subcutaneous approach is minimally invasive, almost painless, does not require general anaesthesia and does not imply blood loss. Secondly, this approach can be repeated and many doses can be administered periodically with no risk for the patients. Thirdly, the clinical use of the remote therapy may be applied as a stand‐alone therapy (alternative to conventional therapies) for patients with increased surgical risk or may represent an adjuvant therapeutic option in combination with conventional therapies for further benefits.

Our experiments bring evidence that subcutaneously transplanted MSC activate hypoxia‐inducible signalling pathways and stimulate local angiogenesis, which are mandatory both for graft survival and for the release of protective molecules at distant sites. It is particularly noteworthy that inflammatory and immune signatures often accompany hypoxia programmes in vivo,^34^ with a significant cross talk between transcription factors that respond to either hypoxia or inflammation.^35^ Thus, a more prudent interpretation of hypoxia as being the only mediator of this process could be warranted. Nevertheless, considering the low level of inflammation produced by small‐dose aggregates and the syngeneic scenario of the transplant procedure (that is associated with no immune responses), we assumed that inflammation had minimal contributions to the local mechanisms activated after MSC transplantation. Still, the increased inflammation associated with high‐dose transplantation may significantly impact the outcome of cell therapy, thus emphasizing the importance of accurately determining the number of transplanted cells in order to achieve positive outcomes.

The extensive lines of evidence that MSC produce a broad repertoire of trophic and immunomodulatory cytokines have highlighted the importance of the MSC secretome in the field of stem cell biology.^16^, ^34^, ^35^, ^36^ Although the secretome composition is varying depending on cell type and tissue origin sources, the common feature is the enrichment in molecules that are associated with the cell survival, angiogenesis process and immune regulatory functions.^16^, ^37^ Our results show that MSC‐based remote therapy produces a broad and transient increase in cytokines in the circulation of transplanted mice, albeit the actual identity of protective factors still remains elusive. The transmission of the protective signal after MSC therapy could be multifactorial, with mediators originating from diverse sources, suggesting that different cellular and molecular mechanisms can be targeted by remote therapy in the attempt to generate therapeutic benefits. In this study, we used the proteome profiler cytokine array in the attempt to identify the putative circulating mediators in the bloodstream of transplanted animals. From the total of 111 tested molecules, 56 of them had at least threefold increased level in the circulation. The cytokine enrichment was clustered in two important groups according to the peak expression. The 1‐day peak grouped cytokines mainly involved in the angiogenesis programme, while the third‐day peak grouped cytokines involved in the broad immunoregulatory and tissue‐repair programmes. However, despite the comprehensive image of the circulating mediators in the bloodstream, a limitation of this approach is that it cannot provide the information regarding the source of the cytokines. Thus, we cannot exclude the possibility that the cytokines whose level had increased, or at least part of them, might be produced by the host organism as a response to the cell transplant. In favour of the second hypothesis is the fact that the same cytokine array applied to the secretome of MSC aggregates assembled in vitro (by hanging‐drop assay) showed only an increase in 20 cytokines (data not shown), thus suggesting the possibility of a more complex transmission of MSC protective signals. We therefore assumed that a fraction of the cytokines detected in the serum of transplanted animals was MSC‐derived and another fraction was host‐derived.

In summary, our study provides evidence that subcutaneous transplantation of MSC could be regarded as an effective therapeutic strategy capable of protecting distant diseased organs. The fact that a large number of MSC transplanted in a single dose were associated with increased inflammation and reduced survival and angiogenesis as opposed to a small number of grafted cells deepens the understanding of cell behaviour after transplantation, and could be key in the development of a safe and non‐invasive cell‐based therapy for distant organ protection. The comprehensive understanding of the cellular and molecular mechanisms of MSC‐based remote therapy could lead to the design of novel intelligent treatments of different diseases.

## CONFLICT OF INTEREST

The authors declare no competing interests.

## AUTHOR CONTRIBUTIONS

**Bogdan Preda:** Conceptualization (equal); Data curation (lead); Formal analysis (equal); Funding acquisition (equal); Investigation (lead); Methodology (lead); Resources (equal); Validation (lead); Writing‐original draft (equal). **Ana Mihaela Lupan:** Formal analysis (supporting); Software (equal). **Carmen Neculachi:** Methodology (supporting). **Ioana Leti:** Methodology (supporting). **Madalina Fenyo:** Methodology (supporting). **Sinziana Popescu:** Methodology (supporting). **Evelyn Rusu:** Methodology (supporting). **Catalina Marinescu:** Methodology (supporting). **Maya Simionescu:** Supervision (supporting); Writing‐original draft (supporting). **Alexandrina Burlacu:** Conceptualization (equal); Formal analysis (equal); Funding acquisition (equal); Supervision (lead); Writing‐review & editing (lead).

## Supporting information

Fig S1‐S7

## Data Availability

The data that support the findings of this study are available from the corresponding author upon reasonable request.
