# Supplementary material for: Evidence of mesenchymal stromal cell adaptation to local microenvironment following subcutaneous transplantation
Source: J Cell Mol Med. 2020 Aug 12;24(18):10889–97. doi: 10.1111/jcmm.15717 (PMC7521285; doi:10.1111/jcmm.15717)
Supplement: Supplementary file 1 — Fig S1‐S7 [file JCMM-24-10889-s001.docx]

**Subcutaneous transplantation of mesenchymal stromal cells promotes systemic release of protective factors by activation of hypoxia and angiogenesis**

Mihai Bogdan Preda, Ana-Mihaela Lupan, Carmen Alexandra Neculachi, Livia Ioana Leti,

Ioana Madalina Fenyo, Sinziana Popescu, Evelyn Gabriela Rusu, Catalina Iolanda Marinescu,

Maya Simionescu, Alexandrina Burlacu*

SUPPLEMENTAL FIGURES

***
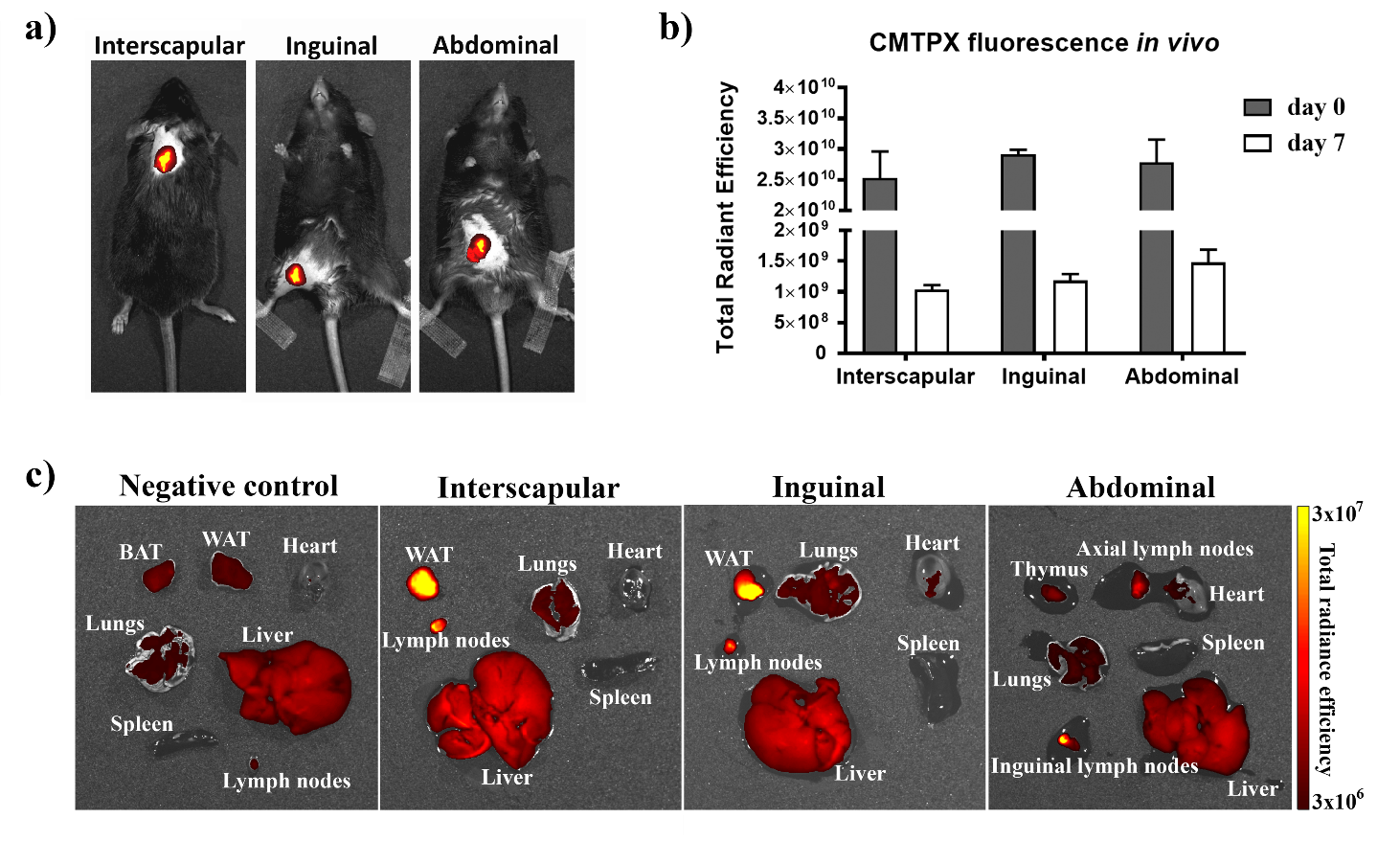
Supplemental figure 1*.** a) Illustration of the three anatomic regions where MSC were subcutaneously transplanted in order to evaluate the effect of adjacent adipose tissue. b) Retention of CMTPX-labelled MSC at injection sites estimated by *in vivo* analysis of the local fluorescent signal at 7 days after cell transplant. No significant difference in the signals are detected between the three anatomic sites. c) *Ex vivo* analysis of cell migration at 7 days after subcutaneous transplantation of CMTPX-labelled MSC determined by the specific fluorescent signal in several organs. Note the presence of detectable, yet dim, signals in lymph nodes and adipose tissue nearby the injection sites.


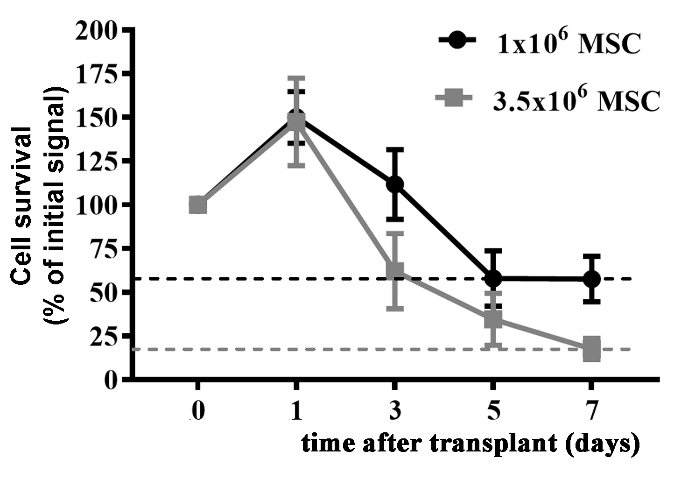


***Supplemental figure 2.*** *In vivo a*ssessment of cell survival measured as the bioluminescent signal (average radiance) coming from the subcutaneously grafted Luc-expressing MSC at different time points after transplantation of low, (1x10^6^ cells) or high cell number (3.5x10^6^ cells). Note that the survival of MSC was considerably higher when low number of MSC were transplanted.


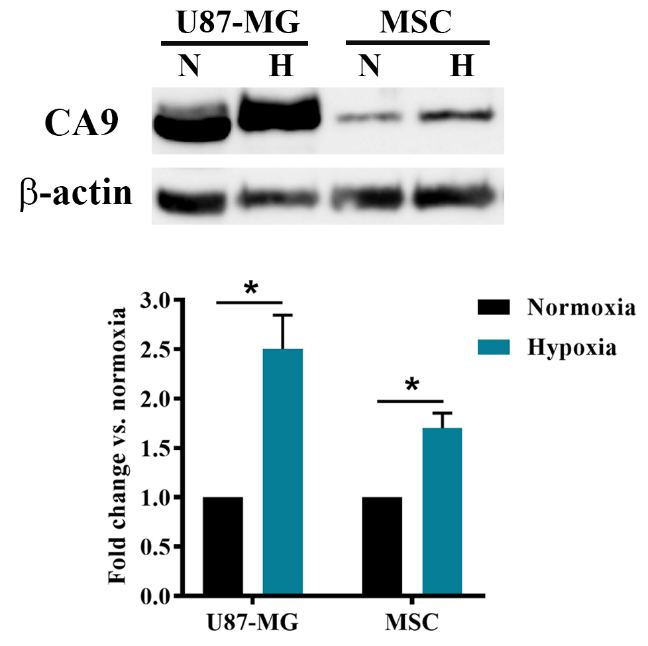


***Supplemental figure 3.*** Protein expression of CA9 in U87-MG cells and MSCs cultivated in normoxic (N, 21% O_2_) or hypoxic (H, 1% O_2_) conditions for 24 hours. Relative quantification was performed by normalization to beta actin and expressed as fold change versus normoxia condition (*p<0.05, Student’s t-test). Note the significant increase in the CA9 level in hypoxic MSC. For original pictures, please refer to Supplemental Figure 7.

**
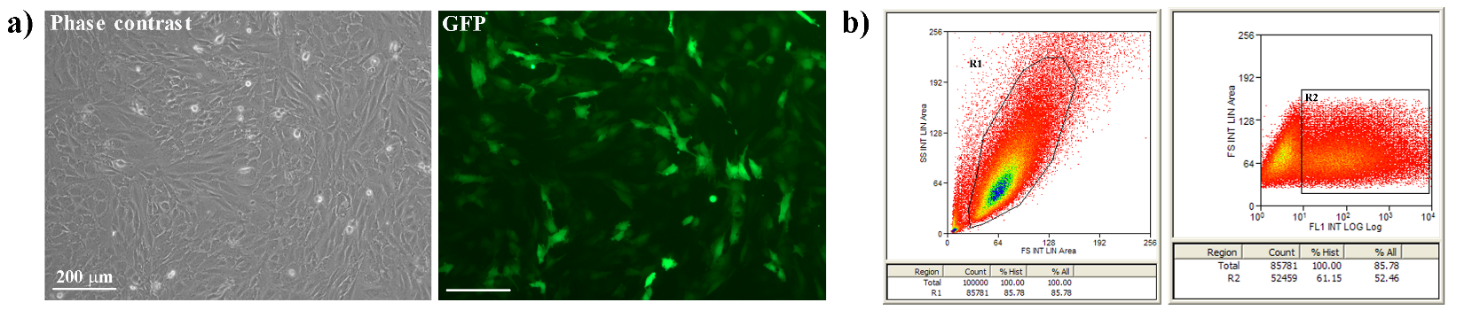
*Supplemental figure 4.*** Assessment of transfection efficiency after electroporation of MSC with pEGFP and analysed 24-hours post-transfection by phase contrast and fluorescence microscopy (a) and flow cytometry (b).


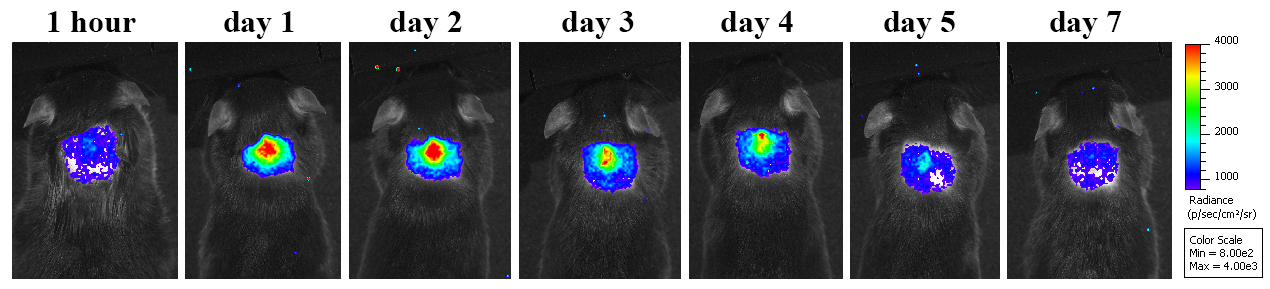


***Supplemental figure 5.*** Time-dependent activation of hypoxia-inducible signalling pathways revealed by the activation of miR-210 promoter in subcutaneously transplanted MSC. Promoter activity of miR-210 was visualized by in vivo imaging analysis of the mouse at different time-points post-injection of MSC transfected to express Luc under the promoter of the hypoxia-inducible miR-210.


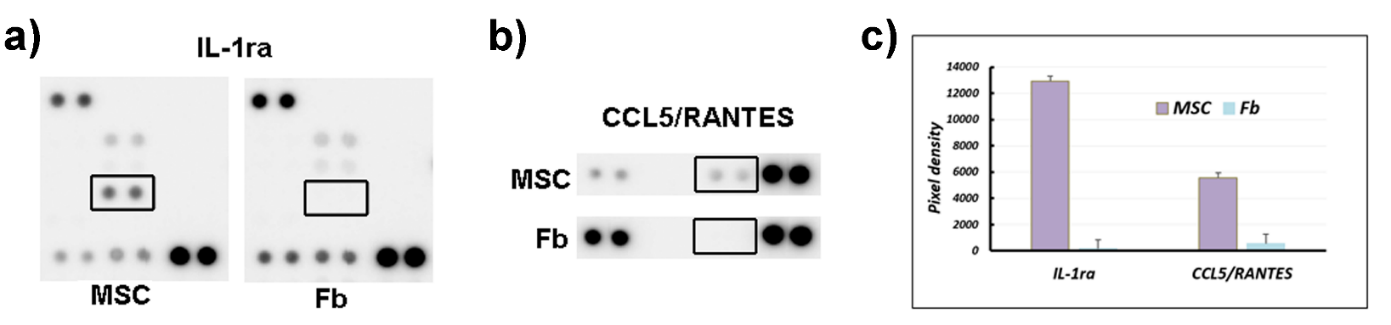


***Supplemental figure 6.*** (a-b) The partial profile of MSC-secreted cytokines versus fibroblasts (Fb)-secreted cytokines in 3D-culture (hanging-drop assay) illustrating IL-1ra (a) and CCL5/RANTES (b) levels in the two cell secretomes. (c) Comparative quantification of the expression level of the two cytokines detected in the secretomes of MSC and Fb.


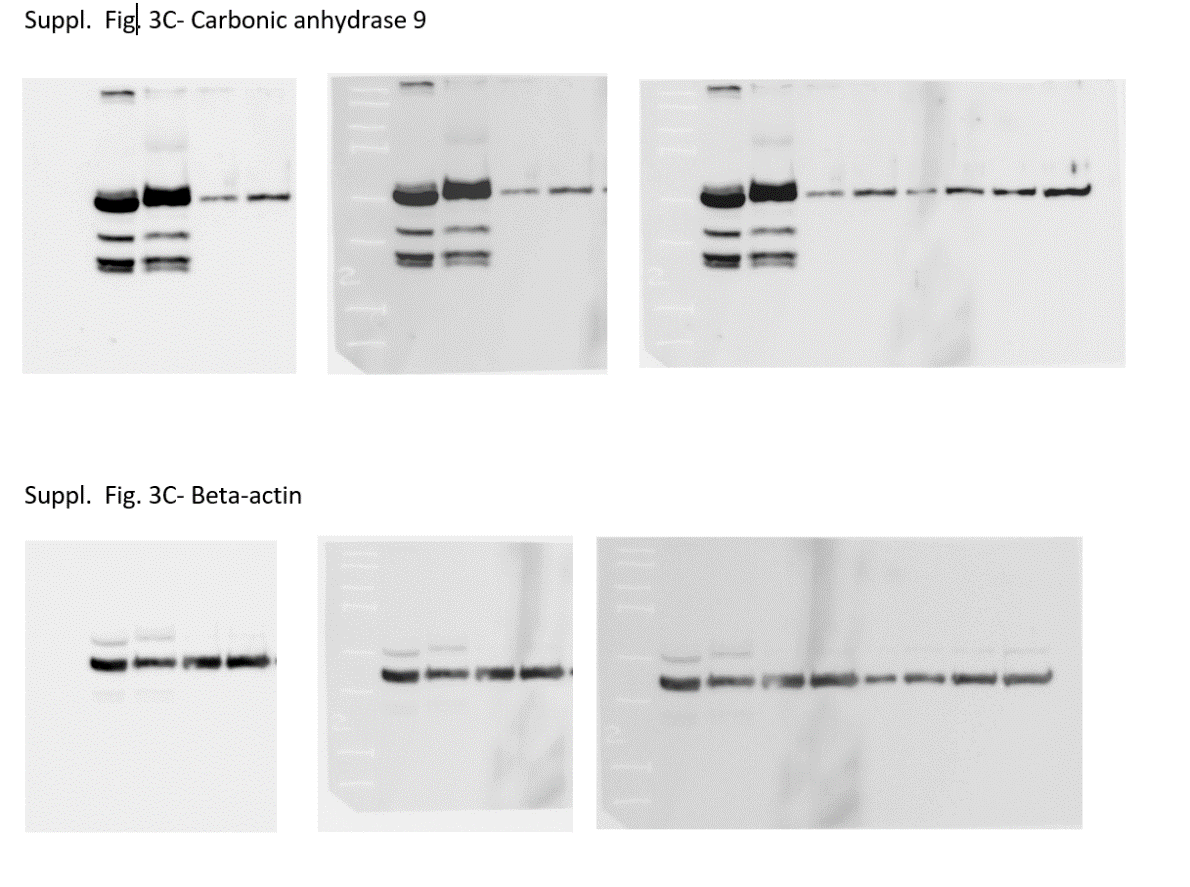


***Supplemental figure 7.***
